# The 3-Phosphoinositide-Dependent Protein Kinase 1 Inhibits Rod Photoreceptor Development

**DOI:** 10.3389/fcell.2018.00134

**Published:** 2018-10-10

**Authors:** Tiaosi Xing, Daniel T. Hass, Samuel S. Zhang, Colin J. Barnstable

**Affiliations:** ^1^Department of Anatomy and Cell Biology, East Carolina University, Greenville, NC, United States; ^2^Department of Neural and Behavioral Sciences, Penn State College of Medicine, Hershey, PA, United States

**Keywords:** photoreceptor, retina, development, IGF-1, PDPK-1, PKC, STAT3, mTORC2

## Abstract

The transition of rod precursor cells to post-mitotic rod photoreceptors can be promoted by extrinsic factors such as insulin-like growth factor 1 (IGF-1), which regulates phosphatidylinositide concentration, and consequently the 3-phosphoinositide-dependent protein kinase-1 (PDPK-1). PDPK-1 is a 63 kDa cytoplasmic kinase that controls cell proliferation and differentiation. In the mouse retina, PDPK-1 and its phosphorylated derivative p-PDPK-1 (Ser241), showed peak expression during the first postnatal (PN) day with a substantial decline by PN7 and in the adult retina. Though initially widely distributed among cell types, PDPK-1 expression decreased first in the inner retina and later in the outer retina. When PDPK-1 is inhibited in neonatal retinal explants by BX795, there is a robust increase in rod photoreceptor numbers. The increase in rods depended on the activity of PKC, as BX795 had no effect when PKC is inhibited. Inhibition of PDPK-1-dependent kinases, such as P70-S6K, but not others, such as mTORC-1, stimulated rod development. The P70-S6K-dependent increase in rods appears to be correlated with phosphorylation of Thr252 and not at Thr389, a substrate of mTORC-1. This pathway is also inactive while PKC activity is inhibited. We also found that inhibition of the kinase mTORC-2, also stimulated by insulin activity, similarly increased rod formation, and this effect appears to be independent of PKC activity. This may represent a novel intracellular signaling pathway that also stimulates photoreceptor development. Consistent with previous studies, stimulation of STAT3 activity is sufficient to prevent any PDPK-1, P70-S6K, or mTORC2-dependent increase in rods. Together the data indicate that PDPK-1 and other intrinsic kinases downstream of IGF-1 are key regulators of rod photoreceptor formation.

## Introduction

Cells within the retina develop in stereotyped order that is well conserved across vertebrates (Young, 1985; La Vail et al., 1991; Stiemke and Hollyfield, 1995). In the rodent retina, these terminal cell divisions occur between embryonic days 10–12 (Barnstable, 1985; Rapaport et al., 2004) and postnatal days 11–12 (Barnstable, 1985; Young, 1985). Rod photoreceptors are the most abundant developing cell of the rodent retina and also among the last to be born, with peak rod generation occurring shortly after birth (Young, 1985; Ezzeddine et al., 1997). The development of these cells requires a combination of intrinsic (Cepko, 1999; Mears et al., 2001; Pinzon-Guzman et al., 2011) and extrinsic signaling molecules (Kelley et al., 1994; Davis et al., 2000) that can regulate the transition of multi-potent stem cells to rod precursors (Lu and Barnstable, 2017) and the differentiation of precursors in to post-mitotic retinal cells (Pinzon-Guzman et al., 2011).

Extrinsic insulin-like growth factor 1 (IGF-1) signaling is one such signal that promotes the transition of rod precursor cells to post-mitotic photoreceptors (Yi et al., 2005; Pinzon-Guzman et al., 2011). Canonically, IGF-1 stimulates phosphatidylinositol-3-kinase (PI_3_K) activity, increasing phosphorylation of the 3′-hydroxyl on intracellular phosphatidylinositol (PIP_3_) (Alessi et al., 1996, 1997; Alessi and Downes, 1998). PIP_3_ is a common second messenger, and one of its major effects is to interact with a central regulator of intracellular signaling, the 3-phosphoinositide-dependent protein kinase-1 (PDPK-1).

3-Phosphoinositide-dependent protein kinase-1 controls cell cycle progression (Nakamura et al., 2008), size (Lawlor et al., 2002), survival (Ito et al., 2009), and differentiation (Dainichi et al., 2016). It is expressed ubiquitously during embryonic stages of mouse development, and PDPK1 null pups do not survive past embryonic day 9.5, indicating the biological importance of this protein (Lawlor et al., 2002). The cellular composition of phospholipids regulates the subcellular localization of PDPK-1, which influences some of its activity. For example, PIP_3_ binding to the PDPK-1 pleckstrin homology domain targets it to the plasma membrane and allows it to phosphorylate protein kinase B (AKT1/PKB) at Thr308 (Alessi et al., 1997; Andjelković et al., 1997; Stephens et al., 1998; Bayascas et al., 2008).

In the adult retina, IGF-1 stimulates classical PI_3_K pathway activity to phosphorylate downstream effectors such as Akt at Ser473. In the PN1 retina, IGF-1 does not appear to have the same effects and decreases Akt (Ser473) phosphorylation. This alteration in IGF-1 signaling is correlated with decreased phosphorylation of the PI_3_K regulatory subunit P55 (*PIK3R3*). This shift in PI_3_K regulation likely decreases PDPK-1 activity and signaling to it’s downstream effectors (Pinzon-Guzman et al., 2011). Under these conditions, non-canonical IGF-1-dependent signaling increases photoreceptor development in a pathway that requires conventional protein kinase C (PKC) isoforms-β1 and -γ. PKC is critical for the activation of downstream tyrosine phosphatases Ship1/2 and to decrease activation of the signal transducer and activator of transcription 3 (STAT3) (Pinzon-Guzman et al., 2010, 2015). STAT3 is a potent regulator of gene expression, and an endpoint in multiple growth factor signaling cascades (Zhang et al., 2004). Activation of STAT3 with cytokines such as CNTF or LIF decreases rod generation (Ezzeddine et al., 1997; Zhang et al., 2004), and infection of *ex vivo* retinas with a dominant negative form of STAT3 prevents basal or CNTF-stimulated inhibition of photoreceptor development (Zhang et al., 2004). While we have defined upstream and downstream intracellular signals that enhance rod development, the intermediate signals in this pathway are not yet clear. Specifically, the mechanism through which PI_3_K inhibition leads to a PKC-dependent decrease in STAT3 activation is not yet known.

As a key intermediate between PI_3_K and downstream signal pathways, we hypothesized that PDPK-1 and its downstream effectors (mTOR, P70-S6K, and PKC) are important signals in rod photoreceptor development. In this study we show that inhibiting PDPK-1 greatly increases the development of rod photoreceptors in retinal explants, in part through PKC-STAT3 signaling and in part through other pathways.

## Materials and Methods

### Ethical Approval

This study was carried out in accordance with the National Research Council’s Guide for the Care and Use of Laboratory Animals (8th edition). The protocol was approved by the Pennsylvania State University College of Medicine Institutional Animal Care and Use Committee.

### Animals

C57B1/6J mice were purchased from Jackson laboratory (Bar Harbor, ME, United States) and housed in a room with an ambient temperature of 25°C, 30–70% humidity, a 12-h light–dark cycle, and *ad libitum* access to rodent chow. All *ex vivo* explant culture experiments used retinal tissue from early postnatal day 1 (PN1) mice.

### Retinal Isolation and Culture

Photoreceptor development was studied using a previously described *ex vivo* retinal explant cultured system (Zhang et al., 2002; Pinzon-Guzman et al., 2011). Briefly, mouse retinas were dissected from PN1 pups and cultured in a defined medium (UltraCulture, Cambrex BioScience) supplemented with 10 μg/mL gentamicin and maintained at 37°C in a 5% CO_2_. Half of this culture medium was changed every 48 h. **Table 1** lists the reagents added to the basal retinal explant culture medium to interrogate the effects of the IGF-1 pathway on photoreceptor development.

**Table 1:** Reagents used in this study.

Reagent	Action	Conc.	Catalog No.	Supplier
Insulin-like growth factor 1 (IGF-1)	IGF-1-R activator	50 μg/mL	I8779	Sigma
BX795	PDPK-1 inhibitor (Feldman et al., 2005)	100 nM	Tlrl-bx7	InvivoGen
Go 7874	PKC inhibitor	50 nM	365252	EMD Millipore
KU0063794	mTORC1/2 inhibitor (García-Martínez et al., 2009)	100 nM	3725	Tocris
PF4708671	P70-S6K inhibitor (Pearce et al., 2010)	200 nM	4032	Tocris
Rapamycin	mTORC1 inhibitor (Sehgal, 2003)	10 nM	9904	Cell signaling
GSK2334470	PDPK-1 inhibitor (Najafov et al., 2011)	3 nM–1 μM	4143	Tocris
Stattic	STAT3 inhibitor	50 μM	573099	EMD Millipore
Leukemia inhibitory factor (LIF)	JAK/STAT3 activator	20 ng/mL	ESG1107	EMD Millipore


Retinal explants for Western blotting were first cultured for 5 h in medium, and then cultured for 30 min in medium supplemented with various reagents at the concentrations listed in **Table 1**, prior to lysis. Explants destined for immunohistochemical studies were cultured for 96 h with or without these reagents prior to fixation.

### Western Blot

Western blots were performed as previously described (Zhang et al., 2004; Pinzon-Guzman et al., 2015). Briefly, retinas were lysed in CytoBuster Protein Extraction Reagent (Novagen) containing EDTA, protease inhibitors (Thermo Scientific), and phosphatase inhibitors (Roche). Samples underwent three freeze-thaw cycles at -20°C, and supernatant was collected after centrifugation. Equal amounts (10–20 μg) of protein were loaded onto polyacrylamide gels (Any kD, Bio-Rad), and separated by standard SDS–PAGE. Proteins were transferred to poly-vinylidene difluoride (PVDF) or nitrocellulose membranes (Bio-Rad) using a standard semi-dry transfer system. Gels were saved for coomassie labeling and normalization to the three histone bands as implemented previously (Popova et al., 2013; Ferreira et al., 2017), and membranes were blocked for 1 h with 5% non-fat dry milk in Tris-buffered saline with 0.1% Tween-20. Membranes were incubated with primary antibody overnight at 4°C, followed by incubation with horseradish peroxidase-conjugated secondary antibodies for 1–2 h at room temperature (See **Table 2** for antibody specificities and dilutions). Semi-quantitative analysis of band intensity was performed using ImageJ^1^.

**Table 2:** Antibodies used in this study.

Target	Host species	Application	Dilution	Catalog no.	Supplier
Akt-1	Rabbit	WB	1:1000	Sc-5298	Santa Cruz
p-AKT-1 (Thr308)	Rabbit	WB	1:1000	2965	Cell signaling
β-Actin (Clone AC-15)	Mouse	WB	1:2500	A1978	Sigma-Aldrich
P70-S6K	Rabbit	WB	1:750	9202	Cell signaling
p-P70-S6K (Thr252)	Rabbit	WB	1:1000	ab59208	Abcam
p-P70-S6K (Thr389)	Rabbit	WB	1:1000	9205	Cell signaling
PCNA (PC10)	Mouse	IF	1:1000	2586	Cell signaling
PDPK-1	Rabbit	WB	1:1000	3062	Cell signaling
PDPK-1	Mouse	IF	1:1000	MA5-15797	Thermo
p-PDPK-1 (Ser241)	Rabbit	IF	1:1000	PA1-14336	Thermo
p-PDPK-1 (Ser241)	Rabbit	WB	1:1000	3438	Cell signaling
Rhodopsin (RetP1)	Mouse	IF	1:100		(29)
STAT3	Rabbit	WB	1:1000	12640S	Cell signaling
p-STAT3 (Tyr 705)	Rabbit	WB	1:500	9131S	Cell signaling
Anti-mouse, HRP-linked	Goat	WB	1:2000–10000	115-035-068	Jackson ImmunoResearch
Anti-rabbit, HRP-linked	Goat	WB	1:2000–10000	1110-35-144	Jackson ImmunoResearch
Anti-mouse, AF488-linked	Goat	IF	1:600	A11001	Life Technologies
Anti-rabbit, AF488-linked	Goat	IF	1:600	A11008	Life Technologies
Anti-mouse, AF594-linked	Goat	IF	1:600	A11005	Life Technologies
Anti-rabbit, AF594-linked	Goat	IF	1:600	A11012	Life Technologies


### Histology and Immunocytochemistry

Immunolabeling of retinal tissue was performed as previously described (Pinzon-Guzman et al., 2011). Briefly, explanted retinas and whole eyes were fixed in 4% paraformaldehyde (Electron Microscopy Sciences, Hatfield, PA, United States) in 1× PBS for 24 h at 4°C. Fixed retinas were either dehydrated through a series of graded ethanol concentrations, embedded in paraffin and sectioned at a 7 μm thickness, or embedded in a 2:1 mixture of 20% sucrose and OCT (Electron Microscopy Sciences), frozen at -20°C, and sectioned at a 5–10 μm thickness. Samples for each experiment were located on the same slide to control for section thickness and assay variability.

Prior to immunohistochemical labeling of deparaffinized or frozen tissue section, we unmasked antigens in a sodium citrate buffer (pH 6.0). Sections were permeabilized in 0.2% Triton-X-100 in PBS, blocked in 5% non-fat milk, and incubated in primary antibodies (See **Table 2** for dilutions) overnight at 4 °C. The following day, sections were washed and incubated in secondary antibody for 3 h, followed by incubation in 1 μg/mL Hochest-33258 for 20 min. After three washes, slides were mounted with 0.5% n-propyl gallate in 1:1 glycerol: PBS. Sections were imaged using an Olympus FLUOVIEW FV1000 confocal microscope. In each set of experiments, acquisition parameters for each antibody were held constant. Fluorescent intensity measurements were derived using the ImageJ measurement tool.

### RNA Isolation and Quantitative Real-Time PCR

RNA was isolated from PN1 retinas using TRIzol (Invitrogen) and purified using RNeasy Mini Kits (Qiagen) as per the manufacturer’s instructions. Final RNA concentration was measured on a Gene Spect III (Hitachi) spectrophotometer. 2 μg RNA samples were reverse transcribed in a total reaction volume of 50 μL using SuperScript III reverse transcriptase (Invitrogen). Quantitative PCR annealing temperatures were set at 60°C for 40 cycles on a Bio-Rad iCycler, followed by a melt curve analysis to confirm primer specificity. Primer sequences are listed in **Table 3**. We quantified relative gene expression using the ΔΔC*_t_* method with GAPDH (QuantiTect; Qiagen) as a reference gene.

**Table 3:** Primers used in this study.

Gene	Forward primer	Reverse primer
Crx	5′-TCAAGAACCGGAGGGCTAAAT-3′	5′-ATAGCTCTGGCCTGATAGGGA-3′
Nrl	5′-GTGCCTCCTTCACCCACCTTCAGTGA-3′	5′ -GCGTGCGGCGCCTCTGCTTCAGCCG-3′
Opsin	5′-TGCTGTTTTCCTTGGCCTTTGG-3′	5′-TCTCTTCAGCATGCCAGGAAGT-3′
Otx2	5′-TCGCCACCTCTACTTTGATAG-3′	5′-AGCCGCATTGGACGTTAG-3′
Sag	5′-GCCATGAGTGTCCTCACC-3′	5′-GGCATGCTGCACTTTCC-3′
Pde6b	5′-CTGACGAGTATGAGGCCAAAG-3′	5′-TAGGCAGAGTCCGTATGCAGT-3′
Rhodopsin	5′-TGCTGTTTTCCTTGGCCTTTG G-3′	5′-TCTCTTCAGCATGCCAGGAAGT-3′


### Statistical Analysis

All statistical analyses were performed using GraphPad Prism. To compare the means of two groups, we used a two-tailed, unpaired *t*-test. For the statistical analysis of more than two groups, we used a one-way ANOVA with a Newman–Keuls *post hoc* test.

## Results

### Developmental Expression of PDPK-1 in Mouse Retina

**FIGURE 1:**
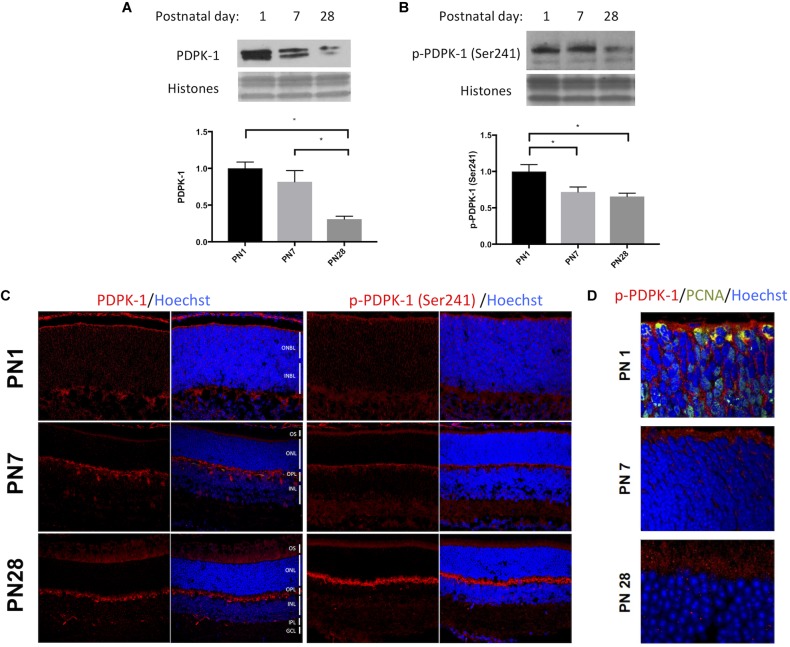
PDPK-1 and p-PDPK-1 expression decline following retinal development. Western blots and corresponding histograms representing **(A)** PDPK-1 and **(B)** p-PDPK-1 Ser241 levels in wild-type PN 1, 7 and adult mouse retinas, normalized to the three histone bands on a coomassie stain of the gel. **(C)** Immunofluorescent labeling of PDPK-1 and p-PDPK-1 Ser241 (red) and Hoechst-33258 (blue) in 10 μm sections of the postnatal days 1, 7, and 28 wild-type mouse retina. **(D)** The same set of retinas, fluorescently labeled for p-PDPK-1 (red), PCNA (green), and Hoechst-33258 (blue). In this set of images, the objective is focused on the outer neuroblast or nuclear layers. ONBL, outer neuroblast layer; INBL, inner neuroblast layer; OS, outer segments; ONL, outer nuclear layer; OPL, outer plexiform layer; INL, inner nuclear layer; IPL, inner plexiform layer; GCL, ganglion cell layer. *n* = 3 biological replicates/measure ^∗^*p* < 0.05, ^∗∗^*p* < 0.01, and ^∗∗∗^*p* < 0.001.

In the mouse retina, the protein levels of PDPK-1 and its active form, phosphorylated at Ser241, significantly declined between postnatal days (PN) 1, 7, and 28. Relative to PN1, total PDPK-1 protein decreased to 82 ± 15% at PN7 and 31.4% at PN28 (*p* < 0.01; **Figure 1A**). Similarly, p-PDPK-1 (Ser241) levels declined to 72 ± 7% at PN7 (*p* < 0.05) and 65 ± 5% at PN28 (*p* < 0.05; **Figure 1B**). To determine whether PDPK-1 protein was expressed in specific retinal cell populations we labeled retinas fixed at PN1, PN7, or PN28 with antibodies recognizing PDPK-1 or p-PDPK-1 (Ser241). During PN1, both forms of PDPK-1 were widely distributed across the retina, from the outer neuroblast layer to inner neuroblast layer. At PN7, much of the labeling had disappeared and the heaviest labeling of PDPK-1 (but not pPDPK-1) was on cell bodies at the outer edge of the inner nuclear layer and suggests that PDPK-1 is present in horizontal cells (**Figure 1C**). At PN28, outer plexiform labeling remained strong, and the inner plexiform layer was diffusely labeled. We tested whether the p-PDPK-1-expressing population of cells of the outer neuroblast layer were dividing or post-mitotic by labeling fixed PN1 retinas for both proliferating cell nuclear antigen (PCNA; green), and p-PDPK-1 (Ser241) (red; **Figure 1D** and **Supplementary Figure S4**). Though many cells were only labeled with a single marker, there was co-localization (yellow) of PDPK-1 and PCNA in cells proximal to the outer limiting membrane (**Figure 1D**). This shows that pPDPK-1 is present in a portion of dividing cells of the early postnatal retina.

### PDPK-1 Is a Critical Inhibitor of Rod Photoreceptor Differentiation

**FIGURE 2:**
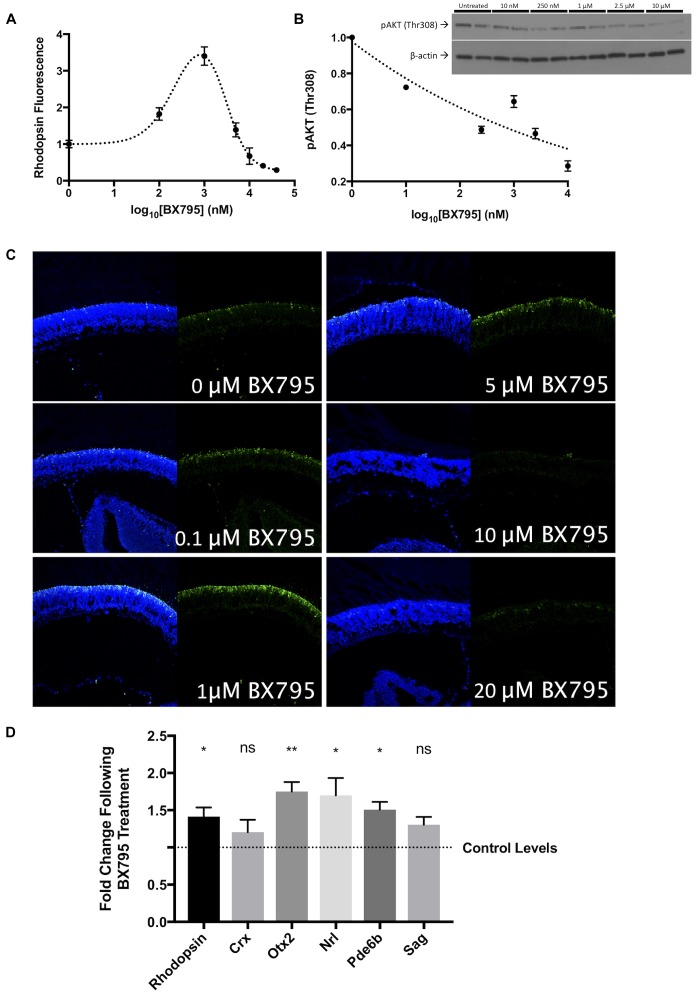
BX795 is a PDPK-1 inhibitor that enhances rod photoreceptor precursor development. In P1 retinal explant cultures, PDPK-1 was inhibited with 0, 0.1, 1, 5, 10, 20, and 40 μM BX795. **(A)** Quantification of rhodopsin labeling intensity as a function of log_10_ (BX795 concentration), relative to untreated controls. **(B)** Protein levels of p-AKT1Thr308 as a function of BX795 concentration. The inset contains an example western blot with duplicate lanes for each concentration of BX795 (*n* = 3). **(C)** Representative images for the curve in A, 96 h retinal explants were sectioned and labeled for rhodopsin (green) and the DNA stain Hoechst-33258 (blue). **(D)** cDNA levels corresponding to the expression of photoreceptor and photoreceptor precursor-related genes. *n* = 3 biological replicates/measure. ^∗^*p* < 0.05 and ^∗∗^*p* < 0.01.

To determine the role of PDPK-1 in photoreceptor development, we treated retinal explants with the competitive inhibitor, BX795 (IC_50_: 6 nM) at concentrations ranging from 10 nM to 20 μM for 96 h. BX795 significantly increased rhodopsin labeling, with a peak effect on rhodopsin (340 ± 25% of control) observed at 1 μM (**Figures 2A,C**). Concentrations of BX795 greater than 1 μM decreased rhodopsin labeling, suggesting off-target or non-specific effects that negatively impact rod development. Notably, we see the same pattern of rhodopsin labeling in explats treated with increasing concentrations of an alternative PDPK-1 inhibitor, GSK2334470 (**Supplementary Figure S1**; Barnstable, 1980). To prevent possible effects, we used 100 nM BX795 in all subsequent experiments, which gives a significant though sub-maximal increase in opsin expression. p-AKT-1 (Thr308) is a PDPK-1 target (Alessi et al., 1997), and as a verification of inhibitor activity, we found a concentration dependent decrease in p-AKT-1 (Thr308) with increasing concentrations of BX795 (**Figure 2B**). To determine whether the action of BX795 was on rod photoreceptors, rather than just on opsin, we tested the expression of a variety of other markers. Though some rod genes (Crx and Sag) showed little change, the expression of others (Nrl, Pde6B, and Otx2) significantly increased, suggesting that the action of BX795 was on more than a single rod gene (**Figure 2D**), and that in our experiments, rhodopsin is a valid proxy for overall photoreceptor development.

### PDPK-1 Inhibits Rod Development in a PKC-Dependent Manner

**FIGURE 3:**
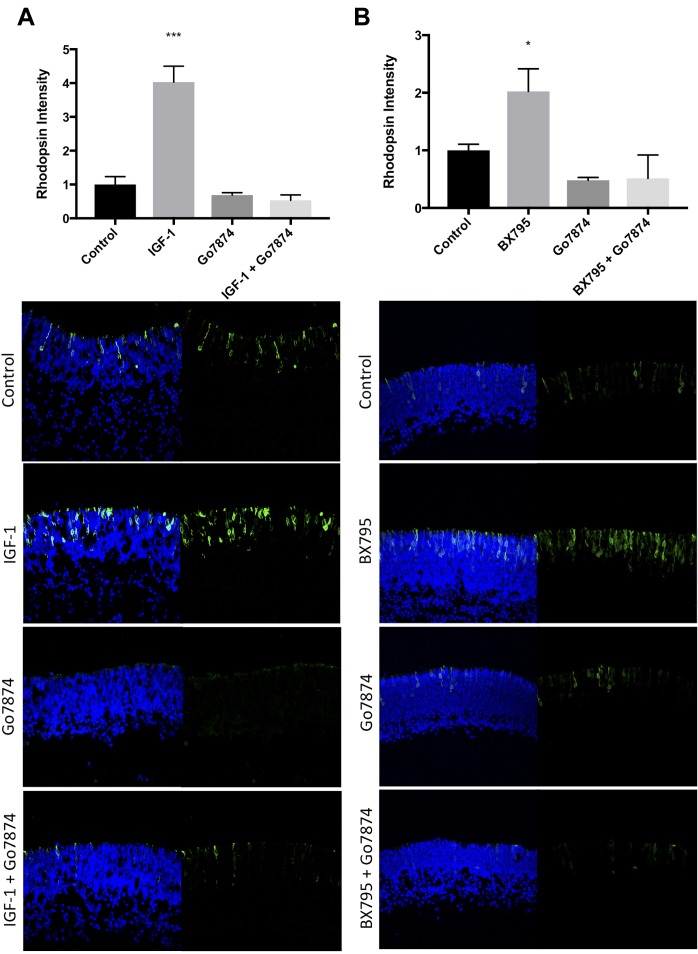
IGF-1 and BX795 enhance rod development in a PKC-dependent manner. Quantification (top) and representative images (bottom) of rhodopsin (green) and Hoechst-33258 (blue) labeled retinal explants following a 96-h exposure to nothing (controls) or **(A)** 50 nM IGF-1, 100 nM Go7874, or both; **(B)** 100 nM Go7874, 100 nM BX795, or both. *n* = 3 biological replicates/measure. ^∗^*p* < 0.05 and ^∗∗∗^*p* < 0.001.

Our previous studies have documented the stimulatory effects of IGF-1 on rod photoreceptor development via a pathway involving activation of PKC (Pinzon-Guzman et al., 2010, 2011). We tested whether the effect of BX795 on rhodopsin was PKC-dependent. As in previous experiments, 50 μg/mL IGF-1 increased rhodopsin labeling to 403 ± 47% (*p* < 0.0001) relative to untreated control explants. Treatment of explants with 50 nM of the PKC inhibitor Go7874 or the combination of IGF-1 and Go7874 decreased rhodopsin labeling to 69 ± 7% (n.s.), and 54 ± 16% (n.s.) of control, respectively (**Figure 3A**).

BX795 also significantly increased rhodopsin levels relative to controls (202 ± 39%, *p* < 0.05) (**Figure 3B**). When Go7874 was co-applied with BX795 there was a decrease in rhodopsin labeling (51 ± 40%) relative to controls, similar to the level of labeling seen with Go7874 alone (48 ± 49%). These data show that the BX795-mediated increase in rhodopsin levels requires PKC activity.

### Inhibition of mTORC-1 Does Not Alter Rod Photoreceptor Development

**FIGURE 4:**
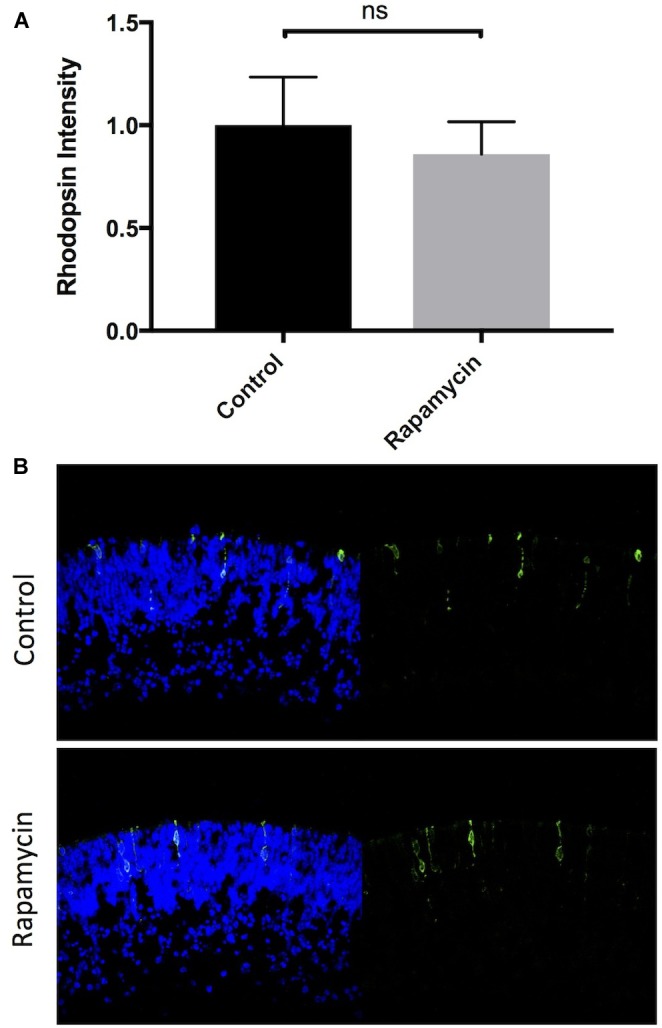
Inhibition of mTORC-1 does not affect rod photoreceptor development. Quantification and representative images of rhodopsin (green) and Hoechst-33258 (blue) labeled retinal explants following a 96-h exposure to nothing (controls) or 10 nM rapamycin.

3-Phosphoinositide-dependent protein kinase-1 lies upstream of AKT1 and thus mTORC-1 signaling (Saxton and Sabatini, 2017). To test whether inhibition of mTORC-1 can influence rhodopsin levels, we treated explants with 10 nM rapamycin for 96 h (Beretta et al., 1996; Sehgal, 2003). This treatment did not significantly alter rhodopsin fluorescence (**Figure 4**), suggesting that mTORC-1 does not normally regulate photoreceptor development. To confirm that the inhibitory effects of rapamycin were still active over similar time periods, we tested the effect of 30 min and 48 h rapamycin treatment on phosphorylation of the mTORC1 target p-P70-S6K (Thr389). Our data suggest that rapamycin is even more effective at decreasing Thr389 levels over this time period than at 30 min, confirming our result (**Supplementary Figure S2**).

### Inhibition of P70-S6K Enhances Rod Differentiation in a PKC Dependent Manner

**FIGURE 5:**
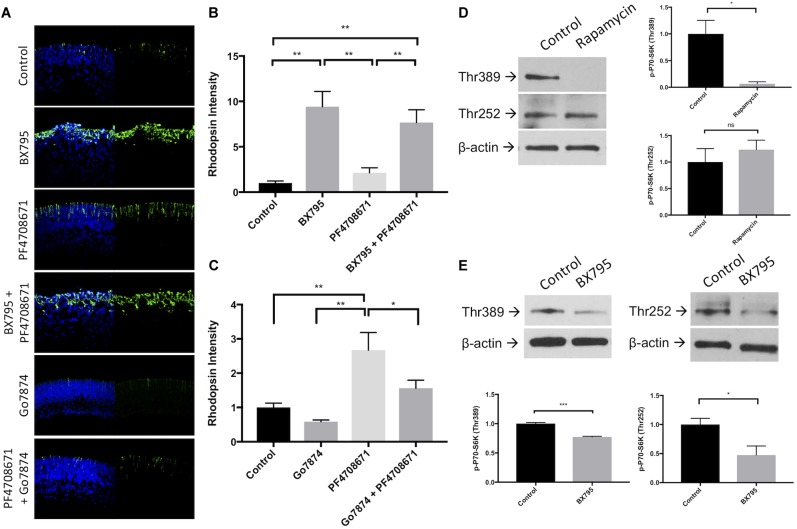
Inhibition of P70-S6K with PF4708671 enhances rod photoreceptor development. **(A)** Representative rhodopsin (green) and Hoechst-33258 (blue) labeling in retinal explants following a 96 h exposure to nothing (control), 100 nM BX795, 200 nM PF4708671, BX795 and PF4708671, 100 nM Go7874, or Go7874 and PF4708671. **(B,C)** Quantification of treatment groups represented in **(A)**. **(D)** Example western blots and densitometric analysis of retinal explants left untreated or treated with 10 nM Rapamycin for 30 min. Lysate from these retinas was labeled with antibodies to detect P70-S6K Thr252, P70-S6K Thr389, and β-actin. **(E)** The same proteins were detected in lysate from retinal explants treated with 100 nM BX795 for 30 min. *n* = 3 biological replicates/measure. ^∗^*p* < 0.05, ^∗∗^*p* < 0.01, and ^∗∗∗^*p* < 0.001.

P70-S6K is major substrate of both PDPK-1 and mTORC-1. Using the P70-S6K inhibitor PF4708671 (IC_50_ = 160 nM) (Pearce et al., 2010), we tested whether inhibition of P70-S6K altered rhodopsin levels. 200 nM PF4708671 significantly increased rhodopsin (242 ± 39%, *p* < 0.01, **Figures 5A,B**), though to a lesser extent than with 100 nM BX795. Concurrent inhibition of PDPK-1 and P70-S6K (941 ± 167%, *p* < 0.01) greatly increased rhodopsin levels, but the increase in rhodopsin was not statistically different from inhibition of PDPK-1 alone (**Figures 5A,B**), suggesting that PDPK-1 and P70-S6K may work in the same pathway to regulate photoreceptor development.

To test more directly if P70-S6K is upstream of PKC, we treated explants with 200 nM PF4708671, 50 nM Go7874, or a combination of these inhibitors for 96 h (**Figures 5A,C**). As shown earlier, rhodopsin levels were reduced in Go7874-treated retinas (59 ± 5% of controls, n.s.). Similarly, rhodopsin was elevated in explants treated with PF4708671 (267 ± 89%, *p* < 0.01). When explants were treated with Go7874 and PF4708671, opsin levels increased to a lower extent than with PF4708671 alone (156 ± 40%, n.s.). These data suggest that while much of the action of P70-S6K is dependent on PKC, the remaining increase in rhodopsin in Go7874 plus PF4708671-treated retinas suggests that part of P70-S6K’s effect on rhodopsin is independent of PKC.

mTORC-1 and PDPK-1 activate P70-S6K kinase activity on separate amino acid residues (mTORC1 phosphorylates Thr389 in mouse and human, whereas PDPK-1 phosphorylates Thr229 in human and Thr252 in mouse) (Burnett et al., 1998; Pullen et al., 1998). Using rapamycin and BX795, we tested whether either or both of these residues was important for retinal development. Application of the mTORC-1 inhibitor rapamycin significantly decreased phosphorylation of P70-S6K Thr389 (*p* < 0.05) but not at Thr252 (**Figure 5D**). On the other hand, BX795 significantly decreased both Thr389 phosphorylation (47% of control, *p* < 0.05) and Thr252 phosphorylation (77% of control, *p* < 0.05; **Figure 5E**). This suggests that the effects of P70-S6K on rod photoreceptor development are particularly dependent on its phosphorylation at Thr252.

### Inhibition of mTORC2 Enhances Rod Differentiation

**FIGURE 6:**
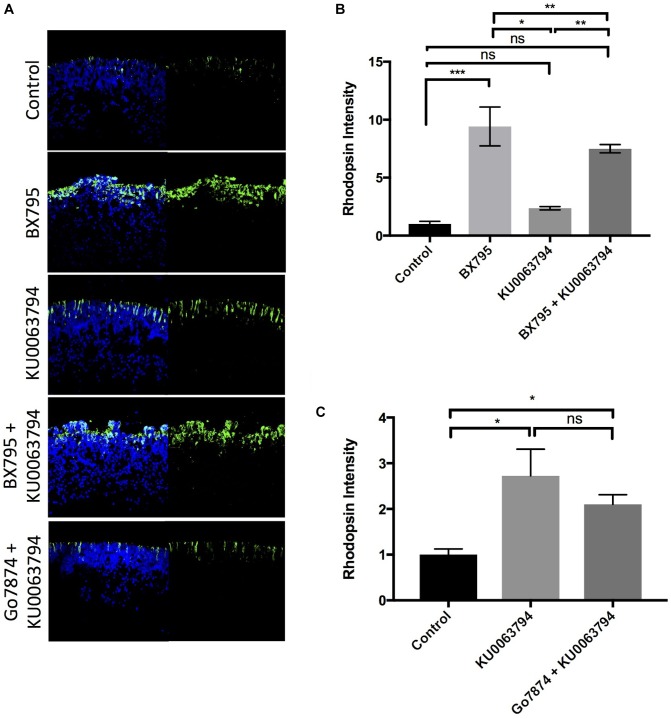
Inhibition of mTORC-1/2 with KU0063794 enhances rod photoreceptor development independently of PKC. **(A)** Example images and **(B,C)** quantification of rhodopsin labeling in retinal explants following a 96-h exposure to nothing (controls) or **(B)** 100 nM KU0063794, 100 nM BX795, or both; **(C)** 100 nM KU0063794, or both. *n* = 3 biological replicates/measure. ^∗^*p* < 0.05, ^∗∗^*p* < 0.01, and ^∗∗∗^*p* < 0.001.

The mTORC2 complex is a regulator of cell growth and metabolism, and the mTORC2 component rictor is a downstream target of P70-S6K (Treins et al., 2010). We hypothesized that like P70-S6K inhibition, mTORC2 inhibition is also able to regulate rod development. There are no known selective inhibitors of mTORC-2, but since mTORC-1 did not influence rod development, we interrogated mTORC-2-dependent effects using the mTORC-1/2 inhibitor KU0063794 (KU; IC_50_ = 10 nM). Treatment of explants with 100 nM KU0063794 enhanced rod photoreceptor development by 236 ± 15% relative to untreated control explants (*p* < 0.05, **Figures 6A,B**). The combined use of KU0063794 and BX795 also stimulated rod development (750 ± 141% of control, *p* < 0.01) approximately to the same extent as BX795 alone (941 ± 167% of control, *p* < 0.001, **Figure 6A**). These data show that mTORC-2 inhibition is likely a component of PDPK-1-inhibition-dependent photoreceptor development.

To determine whether mTORC-2 requires PKC to inhibit rod development, we tested the ability of Go7874 to block the KU0063794 induced increase in rhodopsin levels. As shown in **Figure 6B**, co-application of Go7874 with KU0063794 to retinal explants led to a 210 ± 21% increase in rhodopsin above control, not significantly different from the increase seen with KU0063794 alone (272 ± 58%, *p* < 0.05). These data suggest that the action of mTORC-2 on rod photoreceptor development is independent of PKC.

### STAT3 Activation Prevents BX795-, PF4708671-, and KU0063794-Dependent Rod Formation

**FIGURE 7:**
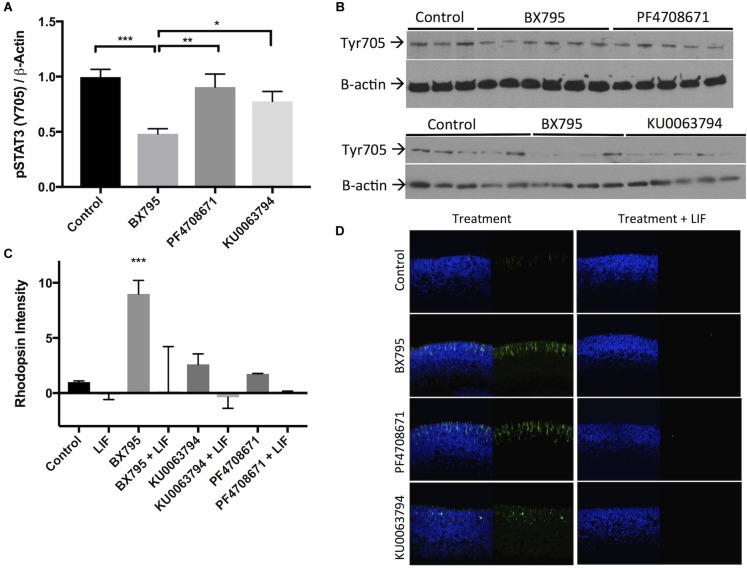
STAT3 activation suppresses rod photoreceptor development, which is prevented by BX795. **(A)** Quantification and **(B)** example western blot demonstrating the effects of 30 min exposures to BX795, PF4708671, and KU0063794 on STAT3 Tyr705 phosphorylation in retinal explants. **(C)** Quantification and **(D)** representative images of rhodopsin (green) and Hoechst-33258 (blue)-labeled retinas following a 96 h exposure to nothing (control), 100 nM BX795, 100 nM KU0063794, 200 nM PF4708671 (left column), or any of the above in combination with 20 ng/mL LIF (right column). ^∗^*p* < 0.05, ^∗∗^*p* < 0.01, and ^∗∗∗^*p* < 0.001.

Activation of STAT3 by phosphorylation of Tyr705 blocks rod photoreceptor development. We hypothesized that inhibitors which increase rhodopsin levels would do so through a pathway that ultimately decrease p-STAT3 Tyr705 levels. 100 nM BX795, but not 100 nM KU0063794 or 200 nM PF4708671, significantly decreased resting p-STAT3 Tyr705 (**Figures 7A,B**). We also tested whether these inhibitors would be sufficient to overcome levels of STAT3 activation stimulated by cytokines such as leukemia inhibitory factor (LIF). We tested the effect of 20 ng/mL LIF on rod photoreceptor development induced by 100 nM BX795, 100 nM KU0063794, or 200 nM PF4708671, and found that LIF-induced STAT3 activity completely halts photoreceptor development, regardless of the presence of BX795, KU0063794, or PF4708671 (**Figures 7C,D**). We also confirmed that the actions of LIF are STAT3 dependent, since while no rods develop in explants treated with 20 ng/mL LIF and 100 nM BX795, rhodopsin labeling is rescued in explants treated with a cocktail of 100 nM BX795, 20 ng/mL LIF, and 50 μM of the STAT3 inhibitor Stattic (**Supplementary Figure S3**). These data suggest STAT3 activity is a prominent regulator of cell fate in the developing retina, perhaps more so than the factors we have studied that stimulate rod development.

## Discussion

Our previous work has demonstrated the presence of an IGF-1-PI_3_K-PKC-Shp-1/2-STAT3 axis in the early postnatal retina that regulates rod photoreceptor development. The present study has demonstrated that PDPK-1, a key downstream effector of the PI_3_K pathway, is part of this axis. Also, we have found that additional pathways downstream of PDPK-1, particularly P70-S6K and mTORC-2, also have a strong inhibitory influence rod photoreceptor development.

Both the normal and active forms of PDPK-1 are expressed in the developing retina and decline during later stages (**Figures 1A,B**). In the early postnatal retina when PDPK-1 levels are greatest, both active and total PDPK-1 are evenly distributed across the tissue (**Figure 1C**), suggesting that PDPK-1 may play a role in the terminal differentiation of multiple retinal cell types. Developing retinal cells extend radial processes across much of the retina. This makes it difficult to measure the co-localization of the cytoplasmic PDPK-1 enzyme and the nuclear proliferation marker PCNA. Nevertheless, we did find some double-labeled cells at the outer margin of the retina, where developing cells undergo mitosis. Interestingly PCNA is cytosolic in these cells, which in granulocytes correlates with differentiation in to neutrophils (Bouayad et al., 2012). We propose that cytosolic PCNA in the retina corresponds to terminally differentiated retinal cells as well. Later in postnatal development, PDPK-1 localized more distinctly to the outer plexiform layer of the retina, with strong staining in horizontal cells (**Figure 1C**), suggesting that it may have a unique function within this cell type. However, the overall decline in PDPK-1 levels suggests that the developmental role of PDPK-1 has concluded by PN7. We did not find any significant perinuclear localization of the active or inactive form of the enzyme, as has been reported by others (Alajajian et al., 2009; Sephton et al., 2009). It is unclear whether this reflects differences in PDPK-1’s role in different tissues or during different developmental stages.

A previously characterized developmental shift in PI_3_K subunits results in IGF-1 inhibiting canonical downstream signaling intermediates such as AKT-1 in the newborn retina, but activating them at later ages (Pinzon-Guzman et al., 2011). As a major regulator of AKT-1, PDPK-1 is also likely inhibited in the early postnatal retina. We modeled this effect by using the PDPK-1 inhibitor BX795 (Feldman et al., 2005). PDPK-1 inhibition caused a significant increase rhodopsin levels (**Figure 2**). Our data showed that this effect is dose dependent up to 1 μM BX795, but the decrease in rhodopsin labeling at greater concentrations suggest that it can affect other proteins at higher concentrations (**Figures 2A,C**). We used 100 nM BX795 in our studies, as it has high specificity for PDPK-1 and a minimal effect on downstream factors such as AKT-1 (**Figure 2B**). While this concentration lessened the magnitude of effect on rhodopsin, we feel that the increased specificity make our results easier to interpret. Additionally, this dose of BX795 was sufficient to increase the expression of multiple rod genes (**Figure 2D**), and we would expect an even greater effect at concentrations of BX795 closer to 1 μM.

Our previous work had shown that the basal or IGF-1 stimulated formation of photoreceptors is dependent on PKC activity (Pinzon-Guzman et al., 2010, 2011). IGF-1 is upstream of PDPK-1 and PKC is downstream of PDPK-1 (Alessi et al., 1997; Le Good et al., 1998) in most biological contexts, and we tested the hypothesis that as with IGF-1, PKC is also downstream of PDPK-1. Our data support this hypothesis, showing that while BX795 and IGF-1 can each significantly increase rhodopsin levels, they are unable to do so when PKC is inhibited (**Figure 3**).

**FIGURE 8:**
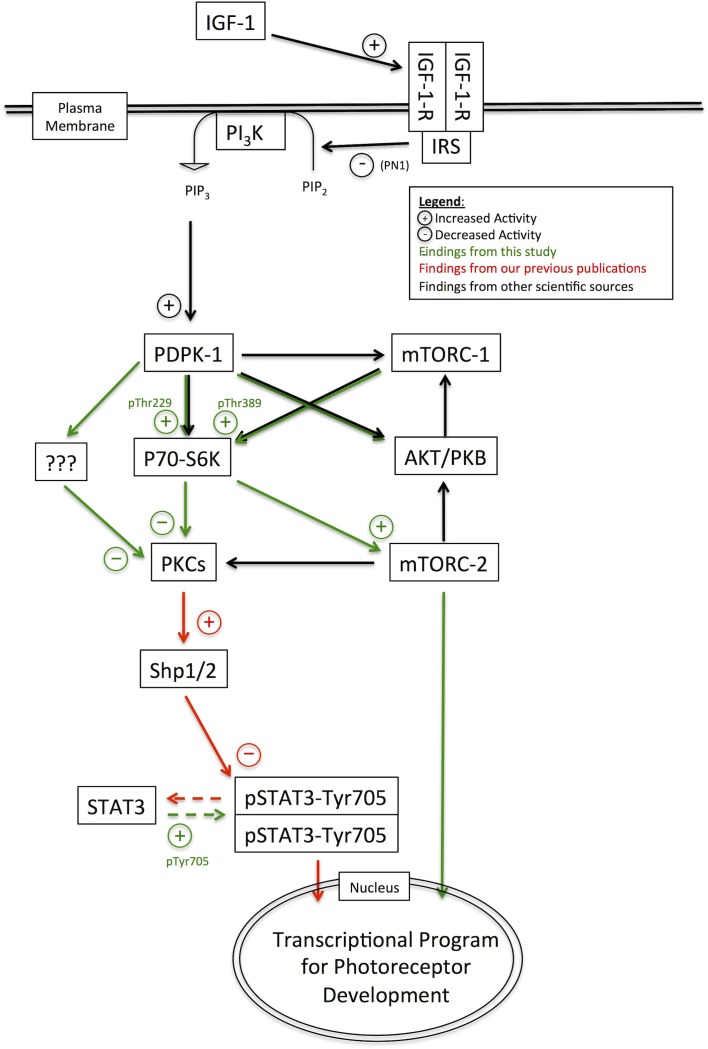
Pathway diagram representing the pathways and interacting factors that control rod photoreceptor development.

3-Phosphoinositide-dependent protein kinase-1 activity leads to the phosphorylation of a number of targets including AKT-1/PKB, mTORC-1, P70-S6K, and mTORC-2. Canonically, PDPK-1 phosphorylates and activates AKT-1/PKB at Thr308, which then indirectly activates mTORC-1. mTORC-1 is has been implicated in the regulation of many diverse processes within cells, from increasing survival to regulating energy homeostasis and cell growth (Guertin and Sabatini, 2007). Despite the ubiquitous number of mTORC-1 targets, mTORC-1 does not appear to regulate rod development (**Figure 4**). This was not completely unexpected, as direct inhibition of AKT/PKB also does not appear to regulate rod development (Pinzon-Guzman et al., 2011). However, PDPK-1 and mTORC-1 share several downstream targets (**Figure 8**). One such target that is key in mediating PDPK-1 signaling is P70-S6K, which increases translational activity within a cell by phosphorylating the S6 ribosomal subunit (Pullen et al., 1998; Dufner and Thomas, 1999; Mora et al., 2004; Fenton and Gout, 2011). We inhibited P70-S6K with PF4708671, and noted a significant increase in rhodopsin levels that occurred to a much lesser extend when PKCs were inhibited, suggesting that PKC is downstream of P70-S6K in the determination of rod numbers (**Figure 5**). This finding, while significant, initially seemed to conflict with the lack of effect we observed in rapamycin-treated retinas, as both mTORC-1 and PDPK-1 increase P70-S6K activity. However, these kinases target different activating residues on P70-S6K (Thr389 for mTORC-1 and both Thr389 and Thr252 for PDPK-1). While rapamycin strongly inhibited phosphorylation of Thr389 (without affecting Thr252), BX795 decreased phosphorylation at both sites (**Figure 5**). Comparing these effects, and the ability of P70-S6K inhibition to increase rod photoreceptor development, we suggest that PDPK-1 suppresses photoreceptor development by activating Thr252 of P70-S6K. To our knowledge, this is the first demonstration of the differential effects of phosphorylating these amino acid residues on P70-S6K.

The protein mTOR can be a component of two multi-protein complexes, mTORC-1 and mTORC-2. mTORC-2 is a rapamycin-insensitive protein complex that is responsive to growth factor signaling cascades, and regulates cell proliferation, survival, and metabolism (Saxton and Sabatini, 2017). mTORC-2 can influence rod development, but the stimulatory effect of the mTORC-1/2 inhibitor KU0063794 on rhodopsin levels was insensitive to treatment with Go7874, suggesting that it operates through a pathway that does not involve PKC (**Figure 6**). Like mTORC1, mTORC2 is activated in response to PI_3_K signaling (Wahane et al., 2014), and is a direct target of P70-S6K (Treins et al., 2010), though the functional impact of mTORC2 activation by P70-S6K has not yet been fully explored. It can also phosphorylate AKT-1/PKB or conventional PKC isoforms (Ikenoue et al., 2008), and thus interacts with many different signaling factors that regulate rod development. Overall, mTORC-2 signaling may be one component in a novel pathway that stimulates rod development (**Figure 8**).

Additionally, it may be possible that P70-S6K stimulates rod development through interactions with both PKC- and mTORC2-dependent developmental pathways. If this is the case, it would explain why the rod-stimulating effects of PF4708671 do not appear to be fully silenced by PKC inhibition, though rhodopsin levels in explants treated with PF4708671 were not statistically distinct from those in explants treated with PF4708671 and Go7874. Regardless, it is still remains to be seen specific role this alternate pathway plays in the developmental cellular physiology of photoreceptors.

One central characteristic of rod photoreceptor development is that it is fully inhibited by STAT3 activity (Ezzeddine et al., 1997; Zhang et al., 2004). PKC activation promotes rod development in part by decreasing STAT3 Tyr705 phosphorylation through the phosphatases Ship1/2 (Pinzon-Guzman et al., 2015). In the present study, we found that inhibition of PDPK-1, but not of P70-S6K or mTORC-2 effectively decreased STAT3 activation. This suggests that PDPK-1 has multiple targets which inhibit rod development. One major pathway involves PKC and STAT3m as we have previously described. In addition we have found a pathway involving P70-S6K and mTORC2 that can influence rod development without altering STAT3 activity. The PDPK-1 inhibition-dependent decrease in STAT3 activation may explain the more intense increase in rhodopsin levels induced by BX795 treatment relative to KU0063794 and PF4708671. LIF increases STAT3 phosphorylation (Zhang et al., 2005), and we also found that LIF is able to completely overcome the effects of BX795, PF4708671, or KU0063794. This exemplifies, the strength of the STAT3 activating pathway and confirms the overall dominance of STAT3 signaling as a major determinant of rod fate specification. In spite of this, it will be important to understand how mTORC2 and P70-S6K can influence the formation of rod photoreceptors, and possibly other retinal cell types.

It will also be important to fully define the developmental role of PDPK-1. There is strong evidence that both its activity and its subcellular localization are highly regulated. Further knowledge concerning the identity of the PDPK-1 regulators, their impact on PDPK-1 localization, and the consequences of PDPK-1 localization on downstream signaling will substantially expand our understanding of how multiple signals trigger or inhibit rod photoreceptor development.

## Author Contributions

TX and DH performed the statistical analysis and wrote the sections of the manuscript. TX wrote the first draft of the manuscript. All authors contributed to conception and design of the study and manuscript revision, read, and approved the submitted version.

## Conflict of Interest Statement

The authors declare that the research was conducted in the absence of any commercial or financial relationships that could be construed as a potential conflict of interest.

## Footnotes

1 http://rsb.info.nih.gov/ij/

## References

[B1] Alajajian B. B. Fletcher L. Isgor E. Jimenez D. F. Digicaylioglu M. (2009). IGF-I regulated phosphorylation and translocation of PDK-1 in neurons. *Neuroreport* 20 579–583. 10.1097/WNR.0b013e328329a41a 19276999

[B2] Alessi D. R. Andjelkovic M. Caudwell B. Cron P. Morrice N. Cohen P. (1996). Mechanism of activation of protein kinase B by insulin and IGF-1. *EMBO J.* 15 6541–6551. 10.1002/j.1460-2075.1996.tb01045.x 8978681 PMC452479

[B3] Alessi D. R. Downes C. P. (1998). The role of PI 3-kinase in insulin action. *Biochim. Biophys. Acta* 1436 151–164. 10.1016/S0005-2760(98)00133-7 9838087

[B4] Alessi D. R. James S. R. Downes C. P. Holmes A. B. Gaffney P. R. Reese C. B. (1997). Characterization of a 3-phosphoinositide-dependent protein kinase which phosphorylates and activates protein kinase Balpha. *Curr. Biol.* 7 261–269. 10.1016/S0960-9822(06)00122-9 9094314

[B5] Andjelković M. Alessi D. R. Meier R. Fernandez A. Lamb N. J. Frech M. (1997). Role of translocation in the activation and function of protein kinase B. *J. Biol. Chem.* 272 31515–31524. 10.1074/jbc.272.50.31515 9395488

[B6] Barnstable C. J. (1980). Monoclonal antibodies which recognize different cell types in the rat retina. *Nature* 286 231–235. 10.1038/286231a0 6157100

[B7] Barnstable C. J. (1985). A molecular view of vertebrate retinal development. *Mol. Neurobiol.* 1 9–46. 10.1007/BF02935263 3077059

[B8] Bayascas J. R. Wullschleger S. Sakamoto K. García-Martínez J. M. Clacher C. Komander D. (2008). Mutation of the PDK1 PH domain inhibits protein kinase B/Akt, leading to small size and insulin resistance. *Mol. Cell. Biol.* 28 3258–3272. 10.1128/MCB.02032-07 18347057 PMC2423167

[B9] Beretta L. Gingras A. C. Svitkin Y. V. Hall M. N. Sonenberg N. (1996). Rapamycin blocks the phosphorylation of 4E-BP1 and inhibits cap-dependent initiation of translation. *EMBO J.* 15 658–664. 10.1002/j.1460-2075.1996.tb00398.x 8599949 PMC449984

[B10] Bouayad D. Pederzoli-Ribeil M. Mocek J. Candalh C. Arlet J. B. Hermine O. (2012). Nuclear-to-cytoplasmic relocalization of the proliferating cell nuclear antigen (PCNA) during differentiation involves a chromosome region maintenance 1 (CRM1)-dependent export and is a prerequisite for PCNA antiapoptotic activity in mature neutrophils. *J. Biol. Chem.* 287 33812–33825. 10.1074/jbc.M112.367839 22846997 PMC3460476

[B11] Burnett P. E. Barrow R. K. Cohen N. A. Snyder S. H. Sabatini D. M. (1998). RAFT1 phosphorylation of the translational regulators p70 S6 kinase and 4E-BP1. *Proc. Natl. Acad. Sci. U.S.A.* 95 1432–1437. 10.1073/pnas.95.4.1432 9465032 PMC19032

[B12] Cepko C. L. (1999). The roles of intrinsic and extrinsic cues and bHLH genes in the determination of retinal cell fates. *Curr. Opin. Neurobiol.* 9 37–46. 10.1016/S0959-4388(99)80005-1 10072376

[B13] Dainichi T. Hayden M. S. Park S.-G. Oh H. Seeley J. J. Grinberg-Bleyer Y. (2016). PDK1 is a regulator of epidermal differentiation that activates and organizes asymmetric cell division. *Cell Rep.* 15 1615–1623. 10.1016/j.celrep.2016.04.051 27184845 PMC4909264

[B14] Davis A. A. Matzuk M. M. Reh T. A. (2000). Activin A promotes progenitor differentiation into photoreceptors in rodent retina. *Mol. Cell. Neurosci.* 15 11–21. 10.1006/mcne.1999.0806 10662502

[B15] Dufner A. Thomas G. (1999). Ribosomal S6 kinase signaling and the control of translation. *Exp. Cell Res.* 253 100–109. 10.1006/excr.1999.4683 10579915

[B16] Ezzeddine Z. D. Yang X. DeChiara T. Yancopoulos G. Cepko C. L. (1997). Postmitotic cells fated to become rod photoreceptors can be respecified by CNTF treatment of the retina. *Development* 124 1055–1067. 9056780 10.1242/dev.124.5.1055

[B17] Feldman R. I. Wu J. M. Polokoff M. A. Kochanny M. J. Dinter H. Zhu D. (2005). Novel small molecule inhibitors of 3-phosphoinositide-dependent kinase-1. *J. Biol. Chem.* 280 19867–19874. 10.1074/jbc.M501367200 15772071

[B18] Fenton T. R. Gout I. T. (2011). Functions and regulation of the 70kDa ribosomal S6 kinases. *Int. J. Biochem. Cell Biol.* 43 47–59. 10.1016/j.biocel.2010.09.018 20932932

[B19] Ferreira R. C. Popova E. Y. James J. Briones M. R. Zhang S. S. Barnstable C. J. (2017). Histone deacetylase 1 is essential for rod photoreceptor differentiation by regulating acetylation at histone H3 lysine 9 and histone H4 lysine 12 in the mouse retina. *J. Biol. Chem.* 292 2422–2440. 10.1074/jbc.M116.756643 28028172 PMC5313111

[B20] García-Martínez J. M. Moran J. Clarke R. G. Gray A. Cosulich S. C. Chresta C. M. (2009). Ku-0063794 is a specific inhibitor of the mammalian target of rapamycin (mTOR). *Biochem. J.* 421 29–42. 10.1042/BJ20090489 19402821 PMC2708931

[B21] Guertin D. A. Sabatini D. M. (2007). Defining the role of mTOR in cancer. *Cancer Cell* 12 9–22. 10.1016/j.ccr.2007.05.008 17613433

[B22] Ikenoue T. Inoki K. Yang Q. Zhou X. Guan K. L. (2008). Essential function of TORC2 in PKC and Akt turn motif phosphorylation, maturation and signalling. *EMBO J.* 27 1919–1931. 10.1038/emboj.2008.119 18566587 PMC2486275

[B23] Ito K. Akazawa H. Tamagawa M. Furukawa K. Ogawa W. Yasuda N. (2009). PDK1 coordinates survival pathways and beta-adrenergic response in the heart. *Proc. Natl. Acad. Sci. U.S.A* 106 8689–8694. 10.1073/pnas.0900064106 19429709 PMC2688981

[B24] Kelley M. W. Turner J. K. Reh T. A. (1994). Retinoic acid promotes differentiation of photoreceptors in vitro. *Development* 120 2091–2102. 7925013 10.1242/dev.120.8.2091

[B25] La Vail M. M. Rapaport D. H. Rakic P. (1991). Cytogenesis in the monkey retina. *J. Comp. Neurol.* 309 86–114. 10.1002/cne.903090107 1894769

[B26] Lawlor M. A. Mora A. Ashby P. R. Williams M. R. Murray-Tait V. Malone L. (2002). Essential role of PDK1 in regulating cell size and development in mice. *EMBO J.* 21 3728–3738. 10.1093/emboj/cdf387 12110585 PMC126129

[B27] Le Good J. A. Ziegler W. H. Parekh D. B. Alessi D. R. Cohen P. Parker P. J. (1998). Protein kinase C isotypes controlled by phosphoinositide 3-kinase through the protein kinase PDK1. *Science* 281 2042–2045. 10.1126/science.281.5385.2042 9748166

[B28] Lu A. Q. Barnstable C. J. (2017). Generation of photoreceptor precursors from mouse embryonic stem cells. *Stem Cell Rev.* 14 247–261. 10.1007/s12015-017-9773-x 29047024

[B29] Mears A. J. Kondo M. Swain P. K. Takada Y. Bush R. A. Saunders T. L. (2001). Nrl is required for rod photoreceptor development. *Nat. Genet.* 29 447–452. 10.1038/ng774 11694879

[B30] Mora A. Komander D. van Aalten D. M. Alessi D. R. (2004). PDK1, the master regulator of AGC kinase signal transduction. *Semin. Cell Dev. Biol.* 15 161–170. 10.1016/j.semcdb.2003.12.022 15209375

[B31] Najafov A. Sommer E. M. Axten J. M. Deyoung M. P. Alessi D. R. (2011). Characterization of GSK2334470, a novel and highly specific inhibitor of PDK1. *Biochem. J.* 433 357–369. 10.1042/BJ20101732 21087210

[B32] Nakamura K. Sakaue H. Nishizawa A. Matsuki Y. Gomi H. Watanabe E. (2008). PDK1 regulates cell proliferation and cell cycle progression through control of cyclin D1 and p27Kip1 expression. *J. Biol. Chem.* 283 17702–17711. 10.1074/jbc.M802589200 18430722

[B33] Pearce L. R. Alton G. R. Richter D. T. Kath J. C. Lingardo L. Chapman J. C. (2010). Characterization of PF-4708671, a novel and highly specific inhibitor of p70 ribosomal S6 kinase (S6K1). *Biochem. J.* 431 245–255. 10.1042/BJ20101024 20704563

[B34] Pinzon-Guzman C. Shaomin M. Zhang S. Barnstable C. J. (2010). Protein kinase C regulates rod photoreceptor differentiation through modulation of STAT3 signaling. *Adv. Exp. Med. Biol.* 664 21–28. 10.1007/978-1-4419-1399-9-3 20237998

[B35] Pinzon-Guzman C. Xing T. Zhang S. S. Barnstable C. J. (2015). Regulation of rod photoreceptor differentiation by STAT3 is controlled by a tyrosine phosphatase. *J. Mol. Neurosci.* 55 152–159. 10.1007/s12031-014-0397-1 25108518 PMC4293205

[B36] Pinzon-Guzman C. Zhang S. S. Barnstable C. J. (2011). Specific protein kinase C isoforms are required for rod photoreceptor differentiation. *J. Neurosci.* 31 18606–18617. 10.1523/JNEUROSCI.2578-11.2011 22171059 PMC3256583

[B37] Popova E. Y. Grigoryev S. A. Fan Y. Skoultchi A. I. Zhang S. S. Barnstable C. J. (2013). Developmentally regulated linker histone H1c promotes heterochromatin condensation and mediates structural integrity of rod photoreceptors in mouse retina. *J. Biol. Chem.* 288 17895–17907. 10.1074/jbc.M113.452144 23645681 PMC3682587

[B38] Pullen N. Dennis P. B. Andjelkovic M. Dufner A. Kozma S. C. Hemmings B. A. (1998). Phosphorylation and activation of p70s6k by PDK1. *Science* 279 707–710. 10.1126/science.279.5351.707 9445476

[B39] Rapaport D. H. Wong L. L. Wood E. D. Yasumura D. LaVail M. M. (2004). Timing and topography of cell genesis in the rat retina. *J. Comp. Neurol.* 474 304–324. 10.1002/cne.20134 15164429

[B40] Saxton R. A. Sabatini D. M. (2017). mTOR signaling in growth, metabolism, and disease. *Cell* 169 361–371. 10.1016/j.cell.2017.03.035 28388417

[B41] Sehgal S. N. (2003). Sirolimus: its discovery, biological properties, and mechanism of action. *Transplant. Proc.* 35(3 Suppl.), 7S–14S. 10.1016/S0041-1345(03)00211-2 12742462

[B42] Sephton C. F. Zhang D. Lehmann T. M. Pennington P. R. Scheid M. P. Mousseau D. D. (2009). The nuclear localization of 3’-phosphoinositide-dependent kinase-1 is dependent on its association with the protein tyrosine phosphatase SHP-1. *Cell. Signal.* 21 1634–1644. 10.1016/j.cellsig.2009.06.010 19591923

[B43] Stephens L. Anderson K. Stokoe D. Erdjument-Bromage H. Painter G. F. Holmes A. B. (1998). Protein kinase B kinases that mediate phosphatidylinositol 3,4,5-trisphosphate-dependent activation of protein kinase B. *Science* 279 710–714. 10.1126/science.279.5351.710 9445477

[B44] Stiemke M. M. Hollyfield J. G. (1995). Cell birthdays in Xenopus laevis retina. *Differentiation* 58 189–193. 10.1046/j.1432-0436.1995.5830189.x 7713326

[B45] Treins C. Warne P. H. Magnuson M. A. Pende M. Downward J. (2010). Rictor is a novel target of p70 S6 kinase-1. *Oncogene* 29 1003–1016. 10.1038/onc.2009.401 19935711

[B46] Wahane S. D. Hellbach N. Prentzell M. T. Weise S. C. Vezzali R. Kreutz C. (2014). PI3K-p110-alpha-subtype signalling mediates survival, proliferation and neurogenesis of cortical progenitor cells via activation of mTORC2. *J. Neurochem.* 130 255–267. 10.1111/jnc.12718 24645666

[B47] Yi X. Schubert M. Peachey N. S. Suzuma K. Burks D. J. Kushner J. A. (2005). Insulin receptor substrate 2 is essential for maturation and survival of photoreceptor cells. *J. Neurosci.* 25 1240–1248. 10.1523/JNEUROSCI.3664-04.2005 15689562 PMC6725974

[B48] Young R. W. (1985). Cell differentiation in the retina of the mouse. *Anat. Rec.* 212 199–205. 10.1002/ar.1092120215 3842042

[B49] Zhang S. S. Fu X. Y. Barnstable C. J. (2002). Tissue culture studies of retinal development. *Methods* 28 439–447. 10.1016/S1046-2023(02)00263-3 12507462

[B50] Zhang S. S. Liu M. G. Kano A. Zhang C. Fu X. Y. Barnstable C. J. (2005). STAT3 activation in response to growth factors or cytokines participates in retina precursor proliferation. *Exp. Eye Res.* 81 103–115. 10.1016/j.exer.2005.01.016 15978261

[B51] Zhang S. S. Wei J. Qin H. Zhang L. Xie B. Hui P. (2004). STAT3-mediated signaling in the determination of rod photoreceptor cell fate in mouse retina. *Invest. Ophthalmol. Vis. Sci.* 45 2407–2412. 10.1167/iovs.04-0003 15223824

