# Supplementary material for: The 3-Phosphoinositide-Dependent Protein Kinase 1 Inhibits Rod Photoreceptor Development
Source: Front Cell Dev Biol. 2018 Oct 10;6:134. doi: 10.3389/fcell.2018.00134 (PMC6191476; doi:10.3389/fcell.2018.00134)
Supplement: Supplementary file 1 [file Data_Sheet_1.docx]

**Supplementary Information**


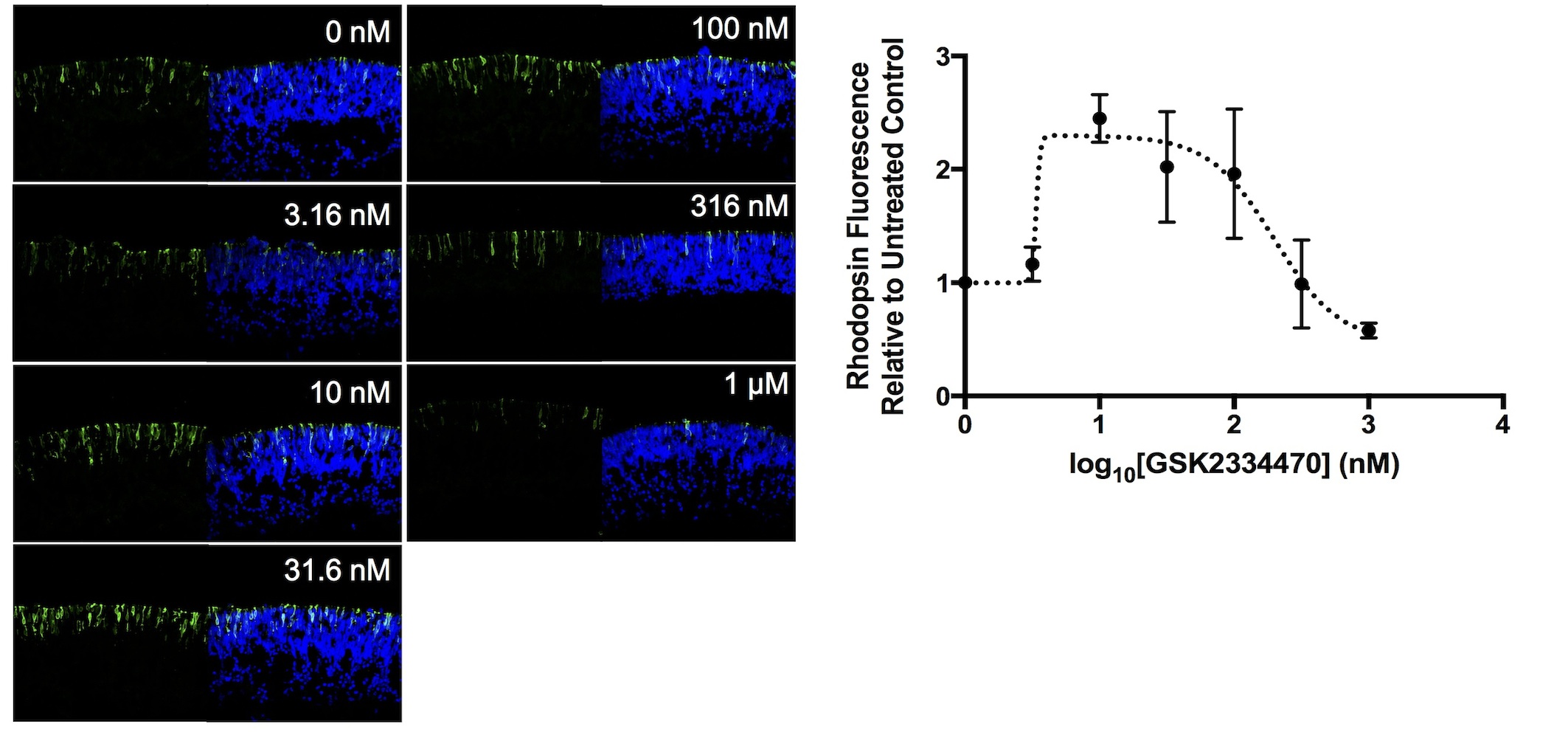


**Figure S1. Low doses of the PDPK-1 inhibitor GSK22334470 increase rhodopsin levels.** (**A**) Fluorescent rhodopsin (green) and Hoechst33442 (blue) labeling and **(B)** corresponding quantification of rhodopsin fluorescence in postnatal day 1 retinal explants treated with increasing concentrations of the PDPK-1 inhibitor GSK22334470. Similarly to the effects of BX795 on the early postnatal retina, GSK22334470 also appears to increase rhodopsin labeling at low doses, and this effect seems to gradually disappear at greater drug concentrations.

**Figure S2. Long-term rapamycin treatment enhances inhibition of p-P70-S6K in retinal explants.** Retinal explants isolated from P1 mice were treated with 10 nM rapamycin for either 30 minutes or 48 hours and immediately frozen in liquid N_2_ prior to downstream applications. Relative to 30 minute rapamycin treatment, 48 hours of exposure provided more complete inhibition of P70-S6K (Thr389) phosphorylation.

**
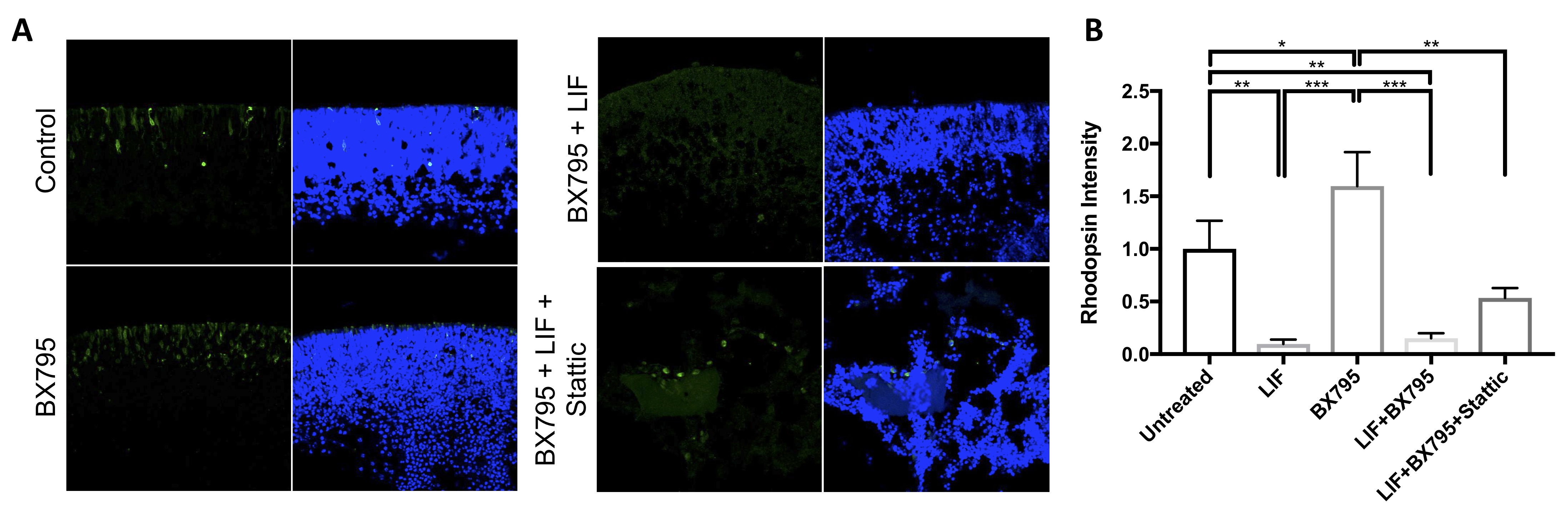
**

**Figure S3. Inhibition of STAT3 dimerization restores rhodopsin expression in LIF-treated retinal explants.**(A) Images and (B) quantification of 96 hour postnatal day 1 retinal explants leftuntreated (control), treated with 100 nM BX795, a combination of 100 nM BX795 and 20 ng/mL LIF, or a combination of 100 nM BX795, 20 ng/mL LIF, and 50 uMStattic. Stattic inhibits STAT3 dimerization regardless of phosphorylation state (Schust et al., 2006). These data show that while the cytokine and Jak activator LIF inhibits rhodopsin expression induced by BX795, a portion of rhodopsin expression is rescued by direct inhibition of STAT3 activity.

**Figure S4. Single staining and merged images for Hoechst, PCNA, p-PDPK-1 in sections of neonatal, PN7, and PN28 retinal explants.** White arrows point towards regions of colocalization between p-PDPK-1 and PCNA.
